# Aspalathin-Enriched Green Rooibos Extract Reduces Hepatic Insulin Resistance by Modulating PI3K/AKT and AMPK Pathways

**DOI:** 10.3390/ijms20030633

**Published:** 2019-02-01

**Authors:** Sithandiwe E. Mazibuko-Mbeje, Phiwayinkosi V. Dludla, Candice Roux, Rabia Johnson, Samira Ghoor, Elizabeth Joubert, Johan Louw, Andy R. Opoku, Christo J. F. Muller

**Affiliations:** 1 Biomedical Research and Innovation Platform, South African Medical Research Council, P.O. Box 19070, Tygerberg 7505, South Africa; pdludla@mrc.ac.za (P.V.D.); candice.roux@mrc.ac.za (C.R.); rabia.johnson@mrc.ac.za (R.J.); samira.ghoor@mrc.ac.za (S.G.); johan.louw@mrc.ac.za (J.L.); christo.muller@mrc.ac.za (C.J.F.M.); 2 Division of Medical Physiology, Faculty of Health Sciences, Stellenbosch University, Private Bag X1, Tygerberg 7505, South Africa; 3 Plant Bioactives Group, Post-Harvest and Agro-Processing Technologies, Agricultural Research Council (ARC), Infruitec-Nietvoorbij, Private Bag X5026, Stellenbosch 7599, South Africa; JoubertL@agric.ac.za; 4 Department of Food Science, Stellenbosch University, Private Bag X1, Matieland 7602, South Africa; 5 Department of Biochemistry and Microbiology, University of Zululand, Private Bag X1001, KwaDlangezwa 3886, South Africa; opokuA@unizul.ac.za

**Keywords:** metabolic syndrome, obesity, insulin resistance, diabetes mellitus, green rooibos extract, therapeutic target, AMPK, PI3K, AKT

## Abstract

We previously demonstrated that an aspalathin-enriched green rooibos extract (GRE) reversed palmitate-induced insulin resistance in C2C12 skeletal muscle and 3T3-L1 fat cells by modulating key effectors of insulin signalling such as phosphatidylinositol-4,5-bisphosphate 3-kinase/protein kinase B (PI3K/AKT) and AMP-activated protein kinase (AMPK). However, the effect of GRE on hepatic insulin resistance is unknown. The effects of GRE on lipid-induced hepatic insulin resistance using palmitate-exposed C3A liver cells and obese insulin resistant (OBIR) rats were explored. GRE attenuated the palmitate-induced impairment of glucose and lipid metabolism in treated C3A cells and improved insulin sensitivity in OBIR rats. Mechanistically, GRE treatment significantly increased PI3K/AKT and AMPK phosphorylation while concurrently enhancing glucose transporter 2 expression. These findings were further supported by marked stimulation of genes involved in glucose metabolism, such as insulin receptor (*Insr*) and insulin receptor substrate 1 and 2 (*Irs1* and *Irs2*), as well as those involved in lipid metabolism, including Forkhead box protein O1 (FOXO1) and carnitine palmitoyl transferase 1 (CPT1) following GRE treatment. GRE showed a strong potential to ameliorate hepatic insulin resistance by improving insulin sensitivity through the regulation of PI3K/AKT, FOXO1 and AMPK-mediated pathways.

## 1. Introduction

Increased consumption of diets high in saturated fats such as palmitate, commonly found in processed and fast foods, contributes to the development of insulin resistance and related conditions, including obesity and type 2 diabetes [1]. In the literature, it is well-documented that obesity induced by a high-fat diet may lead to excessive lipid accumulation in insulin-sensitive tissues, such as skeletal muscle, adipose and the liver [2,3]. Although all the aforementioned insulin-sensitive tissues are important in controlling carbohydrate, lipid, and energy metabolism, liver fat content is more closely correlated with insulin sensitivity [4]. Activation of protein kinase C and subsequent inactivation of insulin receptor attenuate insulin-stimulated insulin receptor substrate 1 and 2 (IRS 1/2) tyrosine phosphorylation, a well-studied mechanism for accelerated hepatic insulin resistance [5,6]. This process is normally initiated by increased lipogenesis, as well as intracellular accumulation of lipid metabolites such as diacylglycerol, and is associated with reduced mitochondrial fatty acid oxidation [7]. Moreover, hepatic insulin resistance suppresses phosphatidylinositol-4,5-bisphosphate 3-kinase/protein kinase B (PI3K/AKT), an important cell survival signaling pathway, leading to reduced glycogen synthase kinase 3 (GSK3) and Forkhead box protein O1 (FOXO1) phosphorylation, which, in turn, results in lower insulin-stimulated liver glycogen synthesis and increased hepatic gluconeogenesis [8].

Recently, studies reported that dietary polyphenols can reduce or prevent insulin resistance and enhance insulin sensitivity. Thus, sources of such polyphenols are of interest as potential therapeutic modulators of glucose and lipid metabolic dysfunction [9,10,11,12]. One such potential source is rooibos, which has gained popularity for its health benefits [13]. In vitro and in vivo data, including that of nonhuman primates, demonstrated that, in addition to ameliorating insulin resistance, rooibos possesses antidiabetic, anti-obesity, anti-inflammatory, and cardio-protective effects [14,15,16,17,18,19]. Furthermore, it is well-documented that beneficial effects of rooibos are attributed to its abundant polyphenolic content [13]. Aspalathin, a dihydrochalcone C-glucoside unique to rooibos, demonstrated various metabolic ameliorative properties [20,21,22,23,24]. For instance, we previously reported that an aspalathin-enriched green rooibos extract (GRE) can ameliorate palmitate-induced insulin resistance in C2C12 muscle and 3T3-L1 cells by modulating key genes involved in energy metabolism and insulin signaling, including PI3K/AKT and AMP-activated protein kinase (AMPK) [15,21]. In this study, we investigated the effects of GRE on lipid-induced hepatic insulin resistance using palmitate-exposed C3A liver cells and a high-fat diet induced obese Wistar rat model (obese insulin resistant (OBIR) rat model). Using these models, the study aimed to elucidate for the first time the cellular mechanisms by which GRE could reverse hepatic insulin resistance.

## 2. Results

### 2.1. GRE Treatment Attenuated Palmitate-Induced Insulin Resistance in C3A Liver Cells

**Figure 1:**
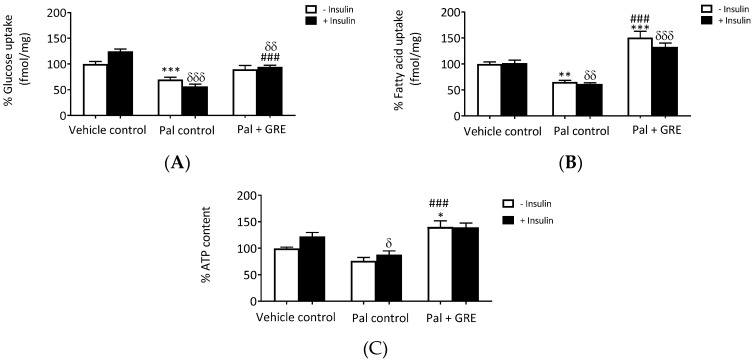
An aspalathin-enriched green rooibos extract (GRE) increased glucose uptake (**A**), palmitate (Pal) uptake (**B**) and ATP content (**C**) in palmitate treated C3A cells. Results are presented as mean ± SEM of three independent experiments. * *p* < 0.05, ** *p* ˂ 0.01, *** *p* < 0.001 versus vehicle control (no insulin), ### *p* < 0.001 versus Pal control (no insulin). δ *p* < 0.05, δδ *p* ˂ 0.01, δδδ *p* < 0.001 versus insulin-stimulated vehicle control.

In this study, we showed that GRE dose dependently increased glucose uptake in normal C3A liver cells to the same level as insulin (Figure S1). We then evaluated the effects of GRE on palmitate-induced insulin resistance in C3A liver cells by measuring glucose and fatty acid uptake. Insulin stimulation increased glucose uptake by ~24 ± 4.3% (*p* < 0.05) in the vehicle control. Palmitate exposure resulted in impaired glucose metabolism by reducing both basal (without insulin) and insulin-stimulated glucose uptake, respectively, by ~30 ± 4.6%, (*p* < 0.001) and ~43 ± 4.7% (*p* < 0.001) (Figure 1A). Similarly, lipid uptake was also impaired, as evident by the reduced fatty acid uptake of ~38 ± 2.3% (*p* < 0.01) in insulin-stimulated C3A liver cells (Figure 1B), confirming that cells were insulin resistant. GRE treatment markedly abrogated the suppressive effect on glucose uptake in insulin-stimulated cells, almost normalizing it to that of vehicle control cells (from 56.3 ± 4.7% to 94.3 ± 3.2% (*p* < 0.001). Moreover, GRE enhanced palmitate fatty acid uptake both in basal (65.0 ± 3.5% to 150.7 ± 12.2%, *p* < 0.001) and insulin-stimulated palmitate treated cells (61.0 ± 2.3% to 132.8 ± 7.6%, *p* < 0.001). Furthermore, GRE increased insulin-stimulated ATP content in palmitate-treated cells from 87.8 ± 7.0% to 139.2 ± 7.3% (*p* < 0.001), respectively, compared to the palmitate control (Figure 1C). However, it was clear that GRE displayed limited effect in improving insulin-sensitizing effects as demonstrated in glucose and fatty acid uptake, as well as ATP experiments (Figure 1).

### 2.2. GRE Reduced Lipid Accumulation and Increased Lipolysis

**Figure 2:**
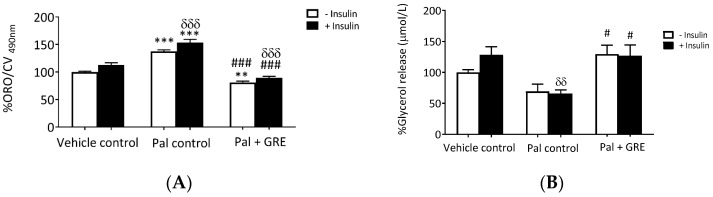
Effect of an aspalathin-enriched GRE on lipid accumulation (**A**) and glycerol release (**B**) in palmitate (Pal) treated C3A cells. Results are presented as mean ± SEM of three independent experiments. ** *p* ˂ 0.01, *** *p* < 0.001 versus vehicle control (no insulin), # *p* < 0.001, ### *p* < 0.001 versus Pal control (no insulin). δδ *p* ˂ 0.01, δδδ *p* < 0.001 versus insulin-stimulated vehicle control.

Our results showed that palmitate treatment increased lipid accumulation by ~37 ± 5.3% (*p* < 0.001) in basal conditions and by ~40 ± 5.0% (*p* < 0.001) in insulin-stimulated cells compared to vehicle control (Figure 2A). This effect was attenuated by GRE treatment with or without insulin from 137.7 to 80.6 ± 5.2% (*p* < 0.001) and 153.3 to 89.2 ± 5.2% (*p* < 0.001) compared to the palmitate control (Figure 2A). Elsewhere, lipolysis was determined by the amount of glycerol released into the media. For this assay, insulin increased lipolysis, albeit not significantly, while palmitate significantly reduced glycerol release (*p* < 0.01). This reduction was reversed (*p* < 0.05) after culturing with GRE with or without insulin compared to the palmitate control (Figure 2B).

### 2.3. GRE Prevented Palmitate-Induced Insulin Resistance through Activation of AKT and AMPK Pathway In Vitro

**Figure 3:**
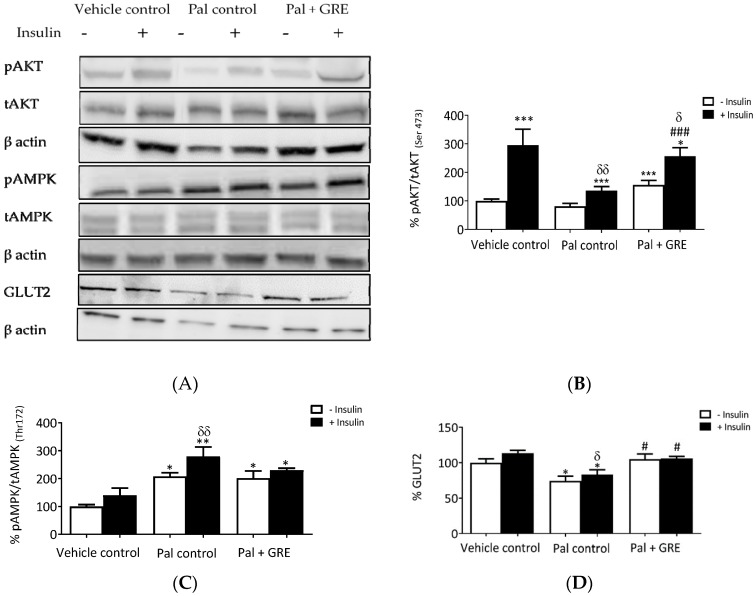
An aspalathin-enriched GRE attenuated palmitate (Pal)-induced insulin resistance in C3A cells. Western blot imaging representation (**A**), Western blot analysis of protein kinase B (AKT) (**B**), AMP-activated protein kinase (AMPK) (**C**), and glucose transporter 2 (GLUT2) protein expression (**D**). Protein expression results are presented as mean ± SEM of three independent experiments. * *p* < 0.05, ** *p* ˂ 0.01, *** *p* < 0.001 versus vehicle control (no insulin), # *p* < 0.05, ### *p* < 0.001 versus Pal control (no insulin). δ *p* < 0.05, δ δ *p* ˂ 0.01 versus insulin stimulated vehicle control.

The potential role GRE plays in modulating key genes and proteins involved in insulin resistance was tested and the results showed that GRE augmented AKT phosphorylation and AMPK gene expression (Figure 3A). Insulin treatment stimulated AKT phosphorylation from 100 ± 6.7% to 296 ± 55.1% (*p* < 0.001), while palmitate exposure significantly reduced insulin-stimulated AKT (Ser 473) activation from 296.0 ± 55.1% to 136.3 ± 13.6% (*p* < 0.001) compared to the vehicle control with insulin (Figure 3B). GRE significantly increased AKT (Ser 473) phosphorylation in the presence of insulin from 136.3 ± 13.6% to 257.1 ± 26.6 % (*p* < 0.05) (Figure 3B). Palmitate enhanced both basal and insulin stimulated AMPK (Thr 172) activation by ~108 ± 12.6% and 179 ± 33.7%, respectively, compared to control cells (*p* < 0.05, *p* < 0.001) (Figure 3C). Interestingly, treating palmitate-exposed cells with GRE also increased AMPK phosphorylation from ~100 ± 25.1% to 140.3 ± 25.3% (*p* < 0.05) under basal conditions when compared to vehicle control (Figure 3C). In addition to reduced phosphorylation of AKT and slight activation of AMPK, C3A liver cells treated with palmitate displayed reduced insulin-stimulated glucose transporter 2 (GLUT2) protein expression from 113.7 ± 4.0% to 83.46 ± 6.4% (*p* < 0.05) (Figure 3D). GRE reversed the palmitate-induced reduction of GLUT2 expression to that of normal control in both basal and insulin-stimulated cells from 74.4 ± 6.7% to 105.1 ± 7.4% (*p* < 0.05) and 83.46 ± 6.7% to 106.0 ± 2.3% (*p* < 0.05), respectively (Figure 3D).

### 2.4. GRE Stimulated Fatty Acid Oxidation-Associated Gene Expression

**Figure 4:**
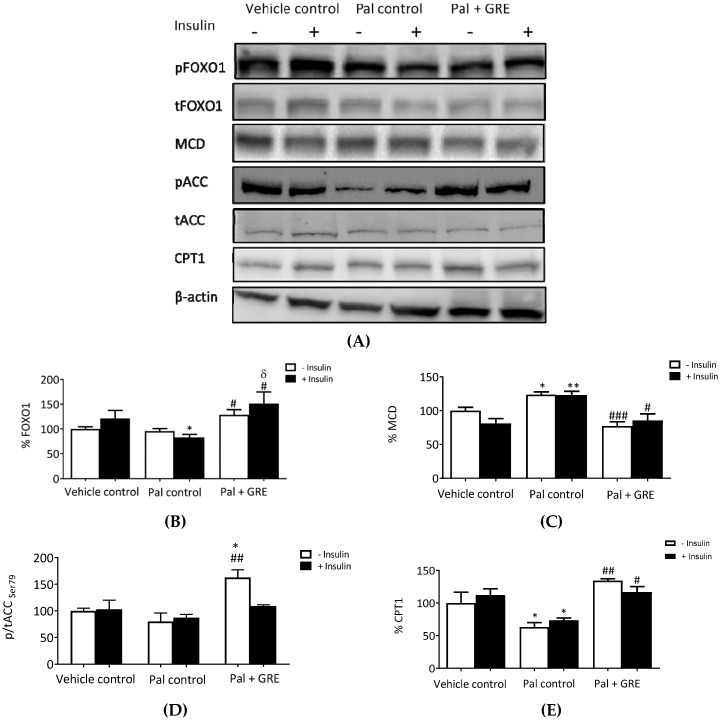
An aspalathin-enriched GRE promoted fatty acid oxidation in palmitate (Pal)-treated C3A cells. Western blot imaging representation (**A**), Western blot analysis of Forkhead box protein O1 (FOXO1) (**B**), malonyl CoA decarboxylase (MCD) (**C**), acetyl CoA carboxylase (ACC) (**D**), and carnitine palmitoyltransferase 1 (CPT1) (**E**). Protein expression results are presented as mean ± SEM of three independent experiments. * *p* < 0.05, ** *p* ˂ 0.01 versus vehicle control (no insulin), # *p* < 0.05, ## *p* ˂ 0.01, ### *p* < 0.001 versus Pal control (no insulin). δ *p* < 0.05 versus insulin-stimulated vehicle control.

This study further evaluated whether GRE could promote fatty acid oxidation in palmitate-induced insulin-resistant liver cells. Palmitate treatment inhibited insulin-stimulated activation of FOXO1 from 121.0 ± 16.2% to 82.7 ± 5.0% (*p* < 0.05) (Figure 4A), and this effect was reversed by treatment with GRE, increasing activation of FOXO1 from 82.7 ± 5.0% to 151.1 ± 23.4% (*p* < 0.05) (Figure 4B). Inhibition of FOXO1 by palmitate was inversely proportional to raised malonyl CoA decarboxylase (MCD) protein expression in both basal and insulin-stimulated C3A liver cells. Treating insulin-resistant C3A liver cells with GRE reduced the palmitate-induced increase of MCD from 123.7 ± 3.9% to 77.2 ± 6.2% (*p* < 0.05) for basal cells and from 123.0 ± 5.5% to 85.3 ± 9.9% (*p* < 0.001) for insulin-stimulated cells (Figure 4C). Palmitate exposure had non significantly reduced the phosphorylation of acetyl CoA carboxylase (ACC) at serine 79, while GRE treatment significantly increased phosphorylation for basal cells by ~80 ± 16.6% (*p* < 0.01), when compared to the palmitate control (Figure 4D). The expression of carnitine palmitoyl transferase 1 (CPT1), the key regulatory enzyme of mitochondrial long-chain fatty acids (LCFA) oxidation, was reduced by ~36, although not significant after palmitate treatment. GRE increased the protein expression of CPT1 from 63.1 ± 7.0% to 134.5 ± 2.6% (*p* < 0.01) for basal cells and from 73.6 ± 3.4% to 116.9 ± 8.2% for insulin-stimulated cells (Figure 4E).

### 2.5. GRE Partially Enhanced Metabolic Profile in OBIR Rat Model

**Figure 5:**
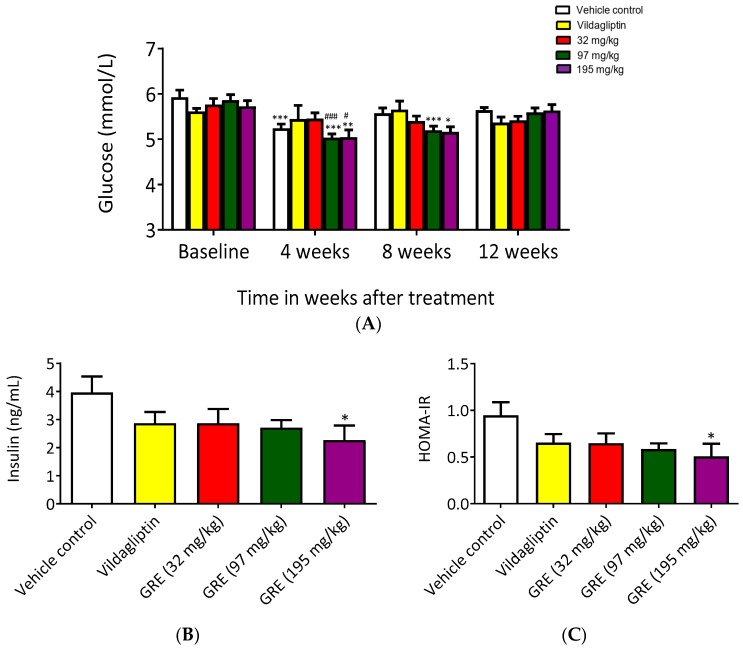
An aspalathin-enriched GRE attenuated insulin resistance in obese insulin resistant (OBIR) Wistar rats. Graphs depict blood glucose concentrations (**A**), insulin concentrations (**B**), and Homeostatic Model Assessment of Insulin Resistance (HOMA-IR) (**C**). OBIR rats were treated daily with GRE. Body weights and blood glucose concentrations were measured at baseline, four weeks, eight weeks, and 12 weeks. The effect of different doses of GRE (32, 97, and 195 mg/kg body weight) was assessed in terms of the reduction in glucose concentrations in comparison to a known HOMA-IR, calculated from fasting glucose and insulin concentrations after 12 weeks of treatment. Results are presented as mean ± SEM of OBIR rats (*n* = 6). * *p* ≤ 0.05, ** *p* < 0.01, *** *p* < 0.001, versus respective baseline values; # *p* ≤ 0.05, ### *p* < 0.001 versus untreated control at 4 weeks after treatment. Baseline indicates measurements taken before treatment.

After establishing the model (Figure S2), we examined the effects of GRE on glucose tolerance and insulin sensitivity by performing oral glucose tolerance tests (OGTTs) and calculating Homeostatic Model Assessment of Insulin Resistance (HOMA-IR) in OBIR rats. Body weights were measured before treatment (baseline) and during and after treatment (four weeks, eight weeks, and 12 weeks). There were no significant differences in body weight or OGTT in any of the treatment groups for the duration of the study (Figure 5A). Similarly, no significant differences were observed with food intake (Figure S3), while the highest doses (97 and 195 mg/kg/day) increased water intake (Figure S4). Consistent with water intake, blood glucose concentrations were affected by GRE at doses of 97 and 195 mg/kg/day reducing glucose concentrations at four and eight weeks compared to their baseline values (Figure 5B). Moreover, a significant reduction in blood glucose concentration was recorded for OBIR rats receiving the highest doses (97 and 195 mg/kg/day) of GRE treatment after week four when compared to the untreated control (Figure 5B). However, after 12 weeks of GRE treatment, no effect on blood glucose concentrations was observed (Figure 5B). The results also showed that, after 12 weeks of treatment, only the highest dose of GRE (195 mg/kg/day) could significantly reduce insulin concentrations and HOMA-IR values when compared to controls (Figure 5C,D).

### 2.6. GRE Improved Insulin Signaling In Vivo

**Figure 6:**
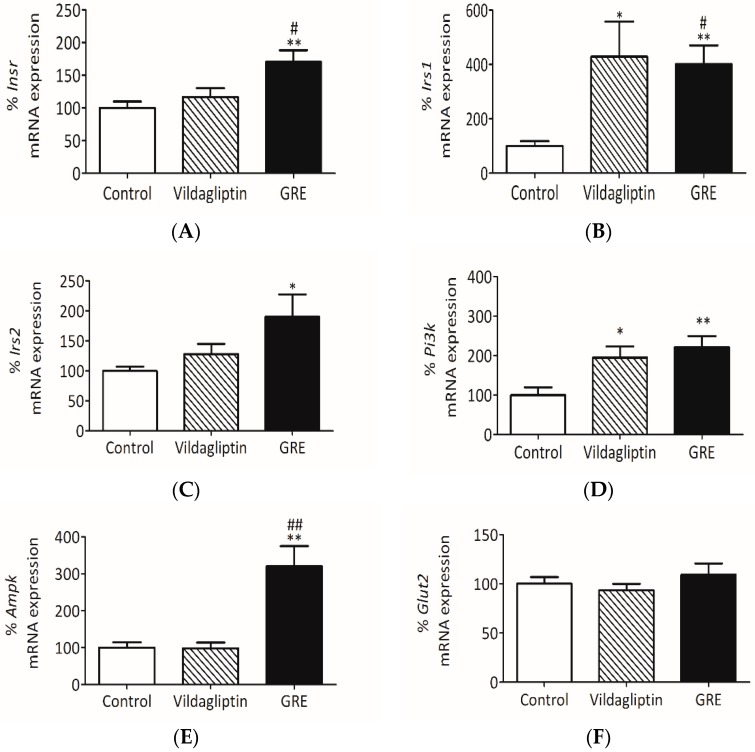
An aspalathin-enriched GRE improved insulin sensitivity by increasing insulin receptor (*Insr*) (**A**), insulin receptor substrate 1 (*Irs1*) (**B**)*,* insulin receptor substrate 2 (*Irs2*) (**C**)*,* phosphatidylinositol-4,5-bisphosphate 3-kinase *Pi3k* (**D**)*,* AMP-activated protein kinase *Ampk* (**E**), and glucose transported 2 (*Glut2*) (**F**) in an obese insulin resistant (OBIR) Wistar rat model. OBIR rats were treated with GRE (195 mg/kg) or vildagliptin (10 mg/kg) for 12 weeks. Total RNA was extracted from liver tissue and gene expression studies were performed by RT-PCR. Data are reported as mean ± SEM of OBIR rats (*n* = 6). * *p* ≤ 0.05, ** *p* ≤ 0.01 versus respective control; # *p* ≤ 0.05, ## *p* ≤ 0.01 versus vildagliptin control.

We further attempted to confirm the mechanism of action by investigating mRNA gene expression involved in insulin signaling in livers of OBIR rats. Vildagliptin, a dipeptidyl peptidase-4 inhibitor class antidiabetic drug, was used as a positive control. GRE significantly increased *Insr* mRNA expression from 100 ± 9.8% to 170.4 ± 7.8% (*p* <0.05) when compared to the normal control (Figure 6A). Moreover, GRE enhanced mRNA gene expression of both *Irs1* and *Irs2* in the liver tissue from 100.0 ± 18.3% to 400.7 ± 68.9% (*p* < 0.05) and from 100.0 ± 6.9% to 190.4 ± 36.9% (*p* < 0.05), respectively (Figure 6B,C). Both vildagliptin and GRE increased *Pi3k* gene expression (120% ± 28.6% (*p* < 0.01) for GRE) (Figure 6D). This was concomitant to raised *Ampk* gene expression affected by GRE when compared to the control (*p* < 0.01) and vildagliptin (*p* < 0.01) (Figure 6E). Moreover, GRE treatment had no effect on *Glut2* mRNA gene expression in vivo (Figure 6F).

## 3. Discussion

Excessive intake of saturated fatty acids is a major causal factor of hepatic lipid accumulation, insulin resistance, and the subsequent development of type 2 diabetes [25]. Therefore, understanding mechanisms that link saturated fatty acid overload or lipid accumulation to impaired insulin signaling within the liver remains crucial in the screening of novel pharmacological and nutritional interventions for their ameliorative properties against insulin resistance and type 2 diabetes mellitus. It is well-known that altered glucose and lipid metabolisms are the main implications of impaired insulin signaling [26]. Therefore, this study used palmitate to induce insulin resistance in C3A cells and a high-fat, high-sugar diet to induce obesity and insulin resistance in Wistar rats. Thereafter, the ameliorative potential of GRE on hepatic insulin resistance was investigated. Furthermore, possible cellular mechanisms by which GRE may ameliorate lipid-induced hepatic insulin resistance were elucidated by exploring downstream signal pathways involved in insulin and AMPK-mediated pathways.

The use of 0.75 mM palmitate was previously shown to effectively reduce insulin sensitivity in muscle cells [27]. This was supported by our previous findings on C2C12 muscle cells and 3T3-L1 adipocytes [15,21], showing that a 0.75 mM palmitate treatment for 16 h induced significant insulin resistance in these cells. Similarly, in the present study, exposing C3A cells to 0.75 mM palmitate resulted in impaired glucose and lipid uptake that affected energy metabolism (ATP content) under both basal (no insulin added) and insulin-stimulated conditions. This was associated with downregulated expression of GLUT2, the main transporter of glucose in the liver. This suggests that insulin resistance can affect the expression of important regulators of glucose uptake and transport. In this in vitro model of insulin resistance, alteration in glucoses uptake and transport were concomitant with enhanced lipid storage and suppressed glycerol release. Interestingly, some of these findings were demonstrated on HepG2 hepatocytes exposed to high concentrations of palmitate [28]. Consistently, protein analysis in our model showed that palmitate exposure reduced phosphorylation of AKT, while slightly increasing that of AMPK. In addition to regulating glucose uptake, phosphorylation of AMPK is also linked with enhanced control of energy generation through β-oxidation [29]. AMPK acts as an energy sensor, regularly responding to cellular energy demands by sensing the balance in AMP:ATP ratio [30]. Therefore, the increased activation of AMPK could be the result of decreasing ATP levels induced by palmitate.

Furthermore, AMPK can regulate β-oxidation by controlling the activities of both ACC and MCD [29]. Malonyl–CoA content is relying on ACC activity for synthesis and MCD for degradation [31]. Mice with mutations in the ACC gene were shown to have raised lipogenesis and reduced FFA oxidation, leading to the progression of insulin resistance, glucose intolerance, and nonalcoholic fatty liver disease [32]. Alternatively, in mice lacking MCD, reduction in fat oxidation is inversely linked with age-associated insulin resistance, while it is directly associated with longevity [33]. In our model of insulin resistance, although ACC phosphorylation was slightly downregulated without significance, a marked increase in the expression of MCD was followed by reduced phosphorylation of FOXO1, as well as impaired AKT and AMPK expression after palmitate exposure. Although activation of AKT is directly correlated with phosphorylation of FOXO1 [34], it was also shown that AMPK phosphorylation increases FOXO1 transcriptional activity, leading to the development of insulin stress resistance and extension in the lifespan of *Caenorhabditis elegans* [35]. Although the function of FOXO1 in hepatic lipid metabolism is not well understood, it was reported that suppression of FOXO1 phosphorylation can hinder insulin signaling through increased gluconeogenesis [36], while increased FOXO1 expression enhances insulin sensitivity [37,38].

Alternatively, an increasing body of knowledge has suggested that regulation of AKT and AMPK can be an effective strategy to prevent insulin resistance and type 2 diabetes [37,38,39]. For example, activation of AKT by certain pharmacological compounds, such as resveratrol, quercetin, and silibinin were shown to be beneficial in combating insulin resistance and associated complications in various experimental models [40,41,42]. Similarly, metformin, which is a preferred first-line antidiabetic drug, is known to improve insulin sensitivity by decreasing hepatic glucose production through the modulation of AMPK [41].

In support of our previous findings in C2C12 skeletal muscle and 3T3-L1 cells [15,21], the current study demonstrated that, in addition to improving energy metabolism through the enhancement of glucose and lipid uptake, GRE significantly increased the phosphorylation of AKT and AMPK, while decreasing gluconeogenesis as observed by increased FOXO1 expression. However, it is of note that the insulin-sensitizing effects of GRE could only be observed at protein level (Figure 3) and no effect was detected biochemical analysis (Figure 1), suggesting further studies are required to investigate such effects. Although the current study showed that the insulin-sensitizing effect of GRE was limited, our results agree with previous findings showing that GRE may present a similar effect to oligonol, a polyphenol rich in lychee fruit, which displayed a strong potential to reduce palmitate-induced fat content while similarly promoting lipolysis in the liver [30]. Moreover, other natural products, such as silibinin, the major active constituent of silymarin, show potential to ameliorate palmitate-induced insulin resistance in C2C12 myotubes through increased insulin-stimulated glucose uptake and via modulation of the IRS1/PI3K/AKT pathway [42]. Elsewhere, findings showed that timosaponin B-II (TB-II), a main ingredient of the traditional Chinese medicine (*Anemarrhena*), as well as resveratrol, can reverse enhanced hepatic dysfunction through the regulation of the IR/IRS1/PI3K/AKT pathway in HepG2 cells and streptozotocin-induced diabetic rats [43,44]. Furthermore, in the liver, cocoa flavonoids have displayed an enhanced potential to strengthen insulin signaling by regulating glucose production through AKT and AMPK in HepG2 cells [45].

Treatment of OBIR rats with GRE for 12 weeks partially confirmed in vitro findings on C3A cells. Here, GRE presented a similar or better effect than Vildagliptin, a known antidiabetic drug, in improving insulin resistance in OBIR rats by reducing elevated HOMA-IR levels and upregulating insulin sensitivity genes, including *Insr*, *Ir1*, *Ir2*, *Ampk*, and *Pi3k*. These results are of interest and support the growing evidence on the ameliorative potential of GRE and its major component (aspalathin) on various metabolic complications associated with the metabolic syndrome. Recent findings show that GRE can improve glucose uptake through the modulation of AMPK in cultured skeletal muscle cells, type 2 diabetic model KK-Ay mice, and streptozotocin-induced diabetic rats [14,18]. In addition to reducing postprandial glucose levels, GRE effectively inhibits carbohydrate metabolizing enzymes in the liver, improves antioxidant status, as well as lowers cholesterol and low-density lipoprotein concentrations [19,46]. Nonetheless, it was also noted that GRE presented a short-term effect in controlling raised blood glucose levels in vivo, suggesting that more studies are required to investigate and confirm the aspect of using GRE over long term period in vivo.

These results are consistent with findings of other natural products showing strong potential to improve insulin signaling by modulating AKT or AMPK [42,43,44,45,46,47,48]. The abundant levels of aspalathin in GRE seem to play a role in enhancing its biological activity, as it was reported to improve various disease condition models, such as hyperglycaemia in obese diabetic *ob/ob* mice, enhance glucose uptake in cardiomyocytes of young rats, and had cardioprotective effects by reducing inflammation in H9c2 cells [20,49,50]. Moreover, treatment of palmitate exposed 3T3-L1 adipocytes with pure aspalathin was able to reverse insulin resistance associated complications [21], and improved glucose and lipid metabolism in cardiomyocytes exposed to high glucose [23].

## 4. Materials and Methods

### 4.1. Reagents and Kits

Human C3A liver cells (ATTC, Cat. No. CRL-10741) were obtained from the American Type Culture Collection (Manassas, VA, USA). GRE, previously designated SB1 which is a solvent-based extract and characterized in terms of its flavonoid profile, was used [14]. Aspalathin, as a major compound, comprised 18.4% of the extract. 2-Deoxy-[^3^H]-d-glucose (DOG) and ^14^C palmitate were obtained from American Radiolabelled Chemicals (St Louis, MO, USA). Bradford and reducing agent compatible and detergent compatible (RC DC) protein assay kits were obtained from Bio-Rad Laboratories (Hercules, CA, USA), while Lonza (Basel, Switzerland) supplied Eagle’s Minimum Essential Medium (EMEM), Dulbecco Modified Eagle’s Medium (DMEM) and the MycoAlert™ mycoplasma detection and ViaLight plus ATP kits. Protease and phosphatase inhibitor tablets were purchased from Roche (South San Francisco, CA, USA). Palmitic acid (C18:0), Vildagliptin, glycerol release assay kit, Oil Red O powder, crystal violet, and other cell culture reagents were obtained from Sigma-Aldrich (St Louis, MO, USA). The 50% Dextrose-Fresenius 50% was from Intramed (Port Elizabeth, South Africa). Mini-PROTEAN TGX Stain-Free™ Precast Gels and Midi Nitrocellulose Transfer Packs were from Biorad Laboratories (Hercules, CA, USA) while, chemiluminescence kits and nitrocellulose membrane were from Amersham Bioscience (Westborough, MA, USA). TRIzol reagent (Invitrogen, Carlsbad, CA, USA), RNeasy Mini kit, RNAlater (Quigen, Hilden, German), and all other chemicals (analytical grade), unless specified, were also obtained from Sigma-Aldrich (St Louis, MO, USA).

### 4.2. Antibodies and Gene Expression Primers

Cell Signalling Technology (Beverly, MA, USA) supplied primary antibodies raised against AKT (cat. # 9272), p-AKT (Ser473) (cat. # 9271), AMPK (cat. # 2532), p-AMPK (Thr172) (cat. # 2531), ACC (cat. # 3662), p-ACC (Ser79) (cat. # 3661), FOXO1 (C29H4) (cat. # 2880), p-FOXO1 (Ser256) (E1F7T) (cat. # 84192) and INSR (β (4B8) (cat. # 3025). Antibodies for GLUT2 (cat. # ab54460), CPT1 (cat. # ab53532) and MCD (cat. # ab95945) were from Abcam (Cambridge, MA, USA). The reference control, β-actin (C4): (sc-47778), and the secondary antibodies, donkey-anti mouse (IgG-HRP: sc-2314) and donkey-anti rabbit (IgG-TR: sc-2784)—horseradish peroxidase, were purchased from Santa Cruz Biotechnology (Dallas, TX, USA). Taqman gene expression assays for *Glut2* (*Hs01086390_m1), Irs1*/*2 (Hs00178563_m1*, *Hs00275843_m1), Pi3k (Hs00979691_m1)* and *Ampk (Hs00272166_m1)* were supplied by Applied Biosystems (Foster City, CA, USA).

### 4.3. In Vitro Experiments: Cell Culture, Induction of Insulin Resistance, and Treatment with GRE

C3A liver cells were cultured and sub-cultured in EMEM with essential amino acids, sodium pyruvate, and L-glutamine. Cells were seeded into 24-well plates (55,000 cells/well) for 2-deoxy-[^3^H]-d-glucose and palmitate uptake, 96-well plates (11,000 cells/well) for ATP analysis, and 6-well plates (165,000 cells/well) for protein analysis. The medium was refreshed after two days. C3A cells were confirmed to be free from mycoplasma following the manufacturer’s instructions. We used cells at passages 5–20 for all experiments. A dose study was performed using glucose uptake on normal C3A liver cells to determine effective concentration and insulin (1 µM) was used as a positive control (results presented in Figure S1). For induction of insulin resistance, C3A liver cells, upon confluence were cultured in EMEM containing 2% BSA, 5.5 mM glucose, and 0.75 mM palmitate for 16 h, as previously described [15,21]. Normal controls were included by culturing in EMEM without palmitate. GRE was prepared as previously described [16]. Briefly, the extract was dissolved in 10% dimethyl sulfoxide (DMSO; *w*/*v*) at a stock concentration of 0.1 mg/µL and further diluted in the DMEM culture media without phenol red to yield a final concentration of 10 µg/mL. Final DMSO concentration in the media for GRE was < 0.001% and did not affect cell viability. On the day of the assay, palmitate-induced insulin-resistant C3A liver cells were serum and glucose starved for 30 min before treating with GRE (10 µg/mL) and insulin (1 µM) in DMEM without phenol red containing 8 mM glucose, 2% BSA, and 0.75 mM palmitate for 3 h at 37 °C in 5% CO_2_ and humidified air. Insulin was used to stimulate insulin signaling.

### 4.4. Measurement of 2-Deoxy-[^3^H]-d-Glucose and ^14^C Palmitate Uptake and ATP Contents

^3^H-2-DOG and ^14^C palmitate uptake assays were performed separately by following previously published protocols [14,21]. Briefly, after 3 h culture with relevant treatments, cells were exposed to 0.5 µCi/mL ^3^H-2-DOG or 1 µCi/mL ^14^C palmitate in medium supplemented with GRE for 3 h at 37 °C in 5% CO_2_ and humidified air for determination of glucose and palmitate uptake, respectively. Insulin (1 µM), the positive control, was added during the last 15 min in each experiment. Thereafter, cells were lysed with 0.1 M NaOH before ^3^H-2-DOG or ^14^C activity in the lysate was assessed by liquid scintillation (2220 CA, Packard Tri-Carb series, PerkinElmer, Downers Crove, IL, USA). Intracellular ATP was measured as previously described [21] using the ViaLight plus ATP kit according to the manufacturer’s recommendations. Absorbance was read at 570 nm using a BioTek ELX 800 plate reader (BioTek Instruments Inc., Winooski, VT, USA) and Gen 5 software for data acquisition.

### 4.5. Oil Red O Staining

The Oil Red O assay was performed as previously described [16] with slight modifications. Briefly, after 3 h culture with relevant treatments, cells were fixed with paraformaldehyde (4%) at room temperature for 20 min and washed with PBS three times. Thereafter, cells were incubated with freshly prepared Oil Red O staining solution for 30 min followed by a proper rinse with distilled water three times. Thereafter, cells were immersed in 100% isopropanol for 20–30 s before extraction solution was added, and then transferred to another 96-well plate for measurement of optical density for lipid accumulation at an absorbance of 490 nm using a BioTek ELX 800 plate reader. To normalise Oil Red O data using cell density, cells were stained with 0.01% crystal violet and measured at 570 nm.

### 4.6. Free Glycerol Determination as a Measure of Lypolysis

Lipolysis was evaluated by measuring the amount of glycerol released into the medium after 3 h culture with relevant treatment as previously described in [16]. Briefly, culture media were collected, centrifuged to remove debris, and directly subjected to glycerol measurement using the glycerol release assay kit from Sigma-Adrich, per its manufacturer’s recommendations. Released glycerol was determined using a BioTek ELX 800 plate reader and Gen 5 software for data acquisition. All samples were measured in duplicates. Free glycerol release content was normalized to cell density (crystal violet measured at 570 nm).

### 4.7. Western Blot Analysis

Western blot analysis was performed by modifying a previous protocol [51]. Briefly, 20 or 40 µg heat denatured protein was separated on a Mini-PROTEAN TGX stain-free precast or 10% SDS-PAGE gel and transferred to a PVDF-P membrane. GLUT2 was transferred using Midi Nitrocellulose Transfer Packs membrane. Nonspecific protein labeling was blocked by incubating the membrane in 5% (*w*/*v*) low-fat milk powder in Tris-buffered saline with Tween 20 at room temperature for 2 h. Membranes were then labelled overnight at 4°C with the relevant primary antibodies (p-AKT (Ser 473), AMPK, p-AMPK (Thr172), GLUT2, CPT1, FOXO1, MCD, INSR) and horseradish peroxidase conjugated secondary antibody (β-actin) applied for 1.5 h the following day. Proteins were detected and quantified by chemiluminescence using a Chemidoc-XRS imager and Quantity One 1-D software. Molecular weight band detection was confirmed using image J software (Biorad Laboratories, Hercules, CA, USA), whereas β-actin was used as the reference control.

### 4.8. In Vivo Experiments: Animal Housing and Ethical Clearance

This study was approved by the Ethics Committee for Research on Animals (ECRA) of the South African Medical Research Council (SAMRC) (ECRA code 11/03/H, date 11/03/2012). The study was executed in accordance with the principles and guidelines of the SAMRC as outlined in Guidelines on Ethics for Medical Research: Use of Animals in Research and Training, 2004 (http://www.mrc.ac.za/ethics/ethicsbook3.pdf). Three-week-old weaning male Wistar rats were obtained and housed at the Primate Unit of the SAMRC (Tygerberg, South Africa). The rats were housed individually in wired top and bottom polycarbonate cages, fitted with PerspexTM houses and maintained in a temperature-controlled room of 24–26 °C, humidity of 45–55% with 15–20 air changes per hour and on a 12 h light/dark cycle.

### 4.9. Establishment of an OBIR Rat Model and Administration of GRE

Wistar rats (*n* = 60), 12 rats per group were fed a high-fat, high-sugar diet (40% fat as energy of which 18.3% were saturated fats) and 44% carbohydrates (2.06 Kcal/g). In addition, 30% sucrose and fructose (1:1 *w*/*w*) was added in drinking water ad libitum for 12 weeks to induce obesity and insulin resistance (OBIR), according to an already published model [52]. After 12 weeks on the high-fat diet, the rats were maintained on the diet with daily administration of GRE at different doses (32, 97, and 195 mg/kg) for a further 12 weeks. GRE dosages were extrapolated from human equivalent consumption of one (32 mg/kg), three (97 mg/kg), and six (195 mg/kg) cups of tea daily. The dose was calculated by dividing the soluble solid content of a cup of rooibos infusion (~230 mg/200 mL) [53] by 70 (average human weight) and multiplying with 10 (surface area dosage conversion factor for rats). Vildagliptin, the drug reference control, was administered at a dose of 10 mg/kg, previously shown to be effective [14]. Gelatine-jelly was used as a vehicle to administer GRE and Vildagliptin. Specific dosages, equivalent to the body weight of a specific rat, were aliquoted into molds prior to addition and setting of the jelly.

### 4.10. Parameters Measured in OBIR Rats

Body weights and blood glucose concentrations were determined at baseline and every four weeks, of treatment Blood glucose concentration was determined using a glucometer and strips (OneTouch Select, LifeScan Inc., Milipitas, CA, USA). OGTTs were performed after 12 weeks of treatment. Briefly, after 16 h of fast, rats were gavaged with GRE treatments 1 h (t = −60 min) before administration of a glucose bolus (t = 0 min) at 2 g/kg glucose (50% Dextrose-Fresenius 50%). Plasma glucose concentrations were determined at −60 and at 0, 15, 30, 60, 120, and 240 min, respectively, relative to the glucose bolus (t = 0). Twenty-four-hour water and food intake were determined, in metabolic cages, prior to commencement of treatment with GRE, as well as 12 weeks after treatment with GRE (Figure S5). Upon completion of the study period, all experimental rats were sacrificed under anesthesia after an overnight (16 h) fast and final treatment dose of GRE in jelly 2 h before sacrifice. Blood was collected, HOMO-IR was calculated using plasma glucose levels and insulin levels as previously described [54], liver tissue was removed, weighed, and samples were stored in RNAlater solution (4 °C) to preserve the mRNA integrity. Samples were also frozen in liquid nitrogen and stored at −80 °C until analyses.

### 4.11. RNA Extraction and Quantitative Real-Time PCR

Total RNA was extracted from liver tissue using TRIzol reagent. Briefly, 80–100 mg of liver tissue was homogenized twice using a TissueLyser (Qiagen, Hilden, Germany) at 25 Hz for 2 min. Gene expression was measured by quantitative real-time PCR (qPCR) using the fluorescent TaqMan 5′nuclease assay on an Applied Biosystems 7500 sequence detection system (Thermo ScientificTM, MA, USA). qPCR was performed using cDNA with 2× TaqMan Master Mix and the 20× premade TaqMan gene expression assays (Applied Biosystems) and qPCR conditions were 95 °C for 10 min, followed by 40 cycles of 95 °C for 15 s and 60 °C for 1 min. Gene expression of insulin receptor (Insr, Hs00961557_m1), *Irs1* (Hs00178563_m1), *Irs2* (Hs00275842_s1), *Pi3k* (Hs00979691_m1), *Ampk* (Hs00178903_m1) and glucose transporter 2 (Slc2a2, Hs01096908_m1) were investigated. mRNA was normalised to mouse β-actin (β-actin Hs02742610_g1) and glyceraldehyde-3-phosphate dehydrogenase (*Gapdh*, Hs02786624_g1). Data were analysed using standard curve and 2−delta Ct ABI Standard Quantification (AQ) software (SDS V1.4; Applied Biosystems, Foster City, CA, USA).

### 4.12. Statistical Analysis

In vitro data were expressed as the mean ± SEM of three independent experiments. Significant differences between treatments were calculated using one-way or two-way analysis of variance (ANOVA), followed by a Tukey post hoc test or Student *t*-test for analysis of data obtained with the animal experiment. A *p* < 0.05 was considered significantly different. All statistical analyses were performed using GraphPad Prism 5 software (GraphPad Software Inc. La Jolla, CA, USA).

## 5. Conclusions

The results presented in this study, of an aspalathin-enriched green rooibos extract, provides evidence of its partial beneficial effect in ameliorating metabolic complications associated with insulin resistance and type 2 diabetes. Modulation of glucose and lipid metabolism by targeting the PI3K/AKT and AMPK signaling cascade may possibly provide a strategy for therapeutic intervention in the reversal of insulin resistance and potential treatment of type 2 diabetes and its complications. However, future research directions, which are also important in addressing limitations of the current studywill include testing the therapeutic effects of GRE on an established model of OBIR. Furthermore, investigating phosphorylation/activation status of major proteins will involve insulin independent pathways such as AMPK. This will include inhibitory or gene targeted overexpression experiments to further elaborate on the protective effect of GRE against the metabolic syndrome.

## Supplementary Materials

Supplementary materials can be found at http://www.mdpi.com/1422-0067/20/3/633/s1.

## Abbreviations

AKT	protein kinase B
AMP	Adenosine monophosphate
AMPK	AMP-activated protein kinase
ATP	Adenosine triphosphate
CPT1	Carnitine palmitoyl transferase 1
DMEM	Dulbecco Modified Eagle’s Medium
DMSO	Dimethyl sulfoxide
ECRA	Ethics Committee for Research on Animals
EMEM	Eagle’s Minimum Essential Medium
FOXO1	Forkhead box protein O1
GLUT	Glucose transporter
GRE	Green rooibos extract
GSK3	Glycogen synthase kinase 3
HOMA-IR	Homeostatic Model Assessment of Insulin Resistance
IRS	Insulin receptor substrate
LCFA	Long chain free fatty acids
MCD	Malonyl-CoA decarboxylase
OBIR	Obese insulin resistant
OGTTs	Oral glucose tolerance tests
PI3K	Phosphatidylinositol-4,5-bisphosphate 3-kinase
SAMRC	South African Medical Research Council
