# Supplementary material for: Aspalathin-Enriched Green Rooibos Extract Reduces Hepatic Insulin Resistance by Modulating PI3K/AKT and AMPK Pathways
Source: Int J Mol Sci. 2019 Feb 1;20(3):633. doi: 10.3390/ijms20030633 (PMC6387445; doi:10.3390/ijms20030633)
Supplement: Supplementary file 1 [file ijms-20-00633-s001.pdf]

International Journal of *Molecular Sciences*

MDPI

Article

Aspalathin-Enriched Green Rooibos Extract Reduces Hepatic Insulin Resistance by Modulating PI3K/AKT and AMPK Pathways

Sithandiwe E. Mazibuko-Mbeje [1,2,3,*], Phiwayinkosi V. Dludla [1], Candice Roux [1], Rabia Johnson [1,3], Samira Ghoor [1], Elizabeth Joubert [4,5], Johan Louw [1,6], Andy R. Opoku [6] and Christo J.F. Muller [1,3,6]

[1] Biomedical Research and Innovation Platform, South African Medical Research Council, P.O. Box 19070, Tygerberg, 7505, South Africa; pdludla@mrc.ac.za (P.V.D.); candice.roux@mrc.ac.za (C.R.); rabia.johnson@mrc.ac.za (R.J.); samira.ghoor@mrc.ac.za (S.G.); johan.louw@mrc.ac.za (J.L.); christo.muller@mrc.ac.za (C.J.F.M.)

[2] Institute for Diabetes and Obesity, Helmholtz-Zentrum München, German Research Center for Environmental Health (GmbH), Parkring 13, 85748 Garching, Germany.

[3] Division of Medical Physiology, Faculty of Health Sciences, Stellenbosch University, Private Bag X1, Tygerberg 7505, South Africa.

[4] Plant Bioactives Group, Post-Harvest and Agro-Processing Technologies, Agricultural Research Council (ARC), Infruitec-Nietvoorbij, Private Bag X5026, Stellenbosch, 7599, South Africa.

[5] Department of Food Science, Stellenbosch University, Private Bag X1, Matieland, 7602, South Africa.

[6] Department of Biochemistry and Microbiology, University of Zululand, Private Bag X1001, KwaDlangezwa 3886, South Africa.

* Correspondence: sithandiwe.mazibuko@mrc.ac.za; Tel.: +27-021-938-0341

Supplementary material

In vitro data

Effect of GRE on normal C3A liver cells without palmitate

The effect of the GRE on glucose uptake by C3A liver cells was used to determine the optimal effective concentration *in vitro.* The extracts GRE was freshly made up in cell culture tested sterile water at a stock concentration of 0.1 mg/µl, the stock solution was further diluted to working solutions of (100, 10, 1, 0.1, 0.01, 0.001, 0.0001 µg/mL) thereafter glucose uptake was performed, insulin was used as positive control.

Results

We performed an *in vitro* glucose uptake study to determine the most active concentration for GRE on normal C3A liver cells. Glucose uptake was enhanced in a dose-dependent manner at all concentrations tested by all GRE concentration. This increase was without any cytotoxicity (Data not shown). Therefore, a concentration of 10µM was selected for further assays.

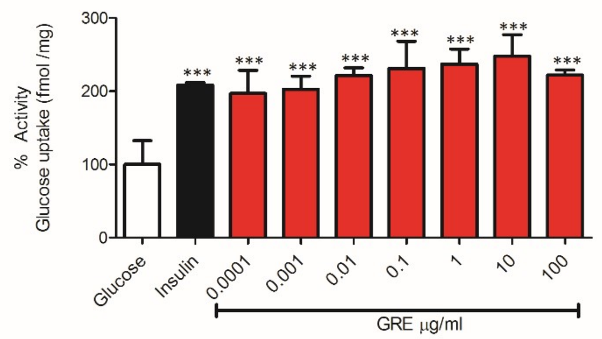

Figure 1S. The effect green rooibos extract (GRE) on glucose uptake in C3A liver cells (B). Cells were cultured in 8 mM glucose in DMEM without phenol red and pyruvate containing log dilutions of GRE for 3 h. Glucose uptake was measured. GRE increased glucose uptake similar insulin at all concentrations. Results are expressed as the mean of three independent experiments expressed relative to control (8 mM glucose) at 100% ± SD; ***p < 0.001.

In vivo models and animal housing

This study was approved by the Ethics Committee for Research on Animals (ECRA) of the South African Medical Research Council (SAMRC) (ECRA 11/03/H). The study was executed in accordance with the principles and guidelines of the SAMRC as outlined in Guidelines on Ethics for Medical Research: Use of Animals in Research and Training, 2004 (http://www.mrc.ac.za/ethics/ethicsbook3.pdf). Three-week-old weaning male Wistar rats were obtained and housed at the Primate Unit of the South African Medical Research Council (Tygerberg, South Africa). The rats were housed individually in wired top and bottom polycarbonate cages, fitted with Perspex houses and maintained in a temperature-controlled room of 24-26°C, humidity of 45-55% with 15-20 air changes per hour and on a 12 h light/dark cycle.

Establishment of OBIR rat model

Rats were divided into 2 groups, control diet group received standard maintenance rat chow (Atlas Animal Feed, Cape Town) and water *ad libitum* (n= 10). High-fat diet (HFD) and 15% sucrose/fructose in drinking water *ad libitum* (n= 10). Diets were administered for three months. Metabolic parameters were measured before termination.

Results

After 3 months of feeding with high fat diet, rats displayed increased body weight and food consumption (Figure 4S A, B, C), while there was no significant change in blood glucose levels between control and high fed diet. However, even though these rats demonstrated ability to

maintain normoglycemia, serum insulin concentrations at three months were increased in high fat diet (Figure 2S D and E). The Homeostatic model assessment-insulin resistance (HOMA-IR) value of the in the high fat diet rats were non-significantly increased when compared to control rats (Figure 2S, F).

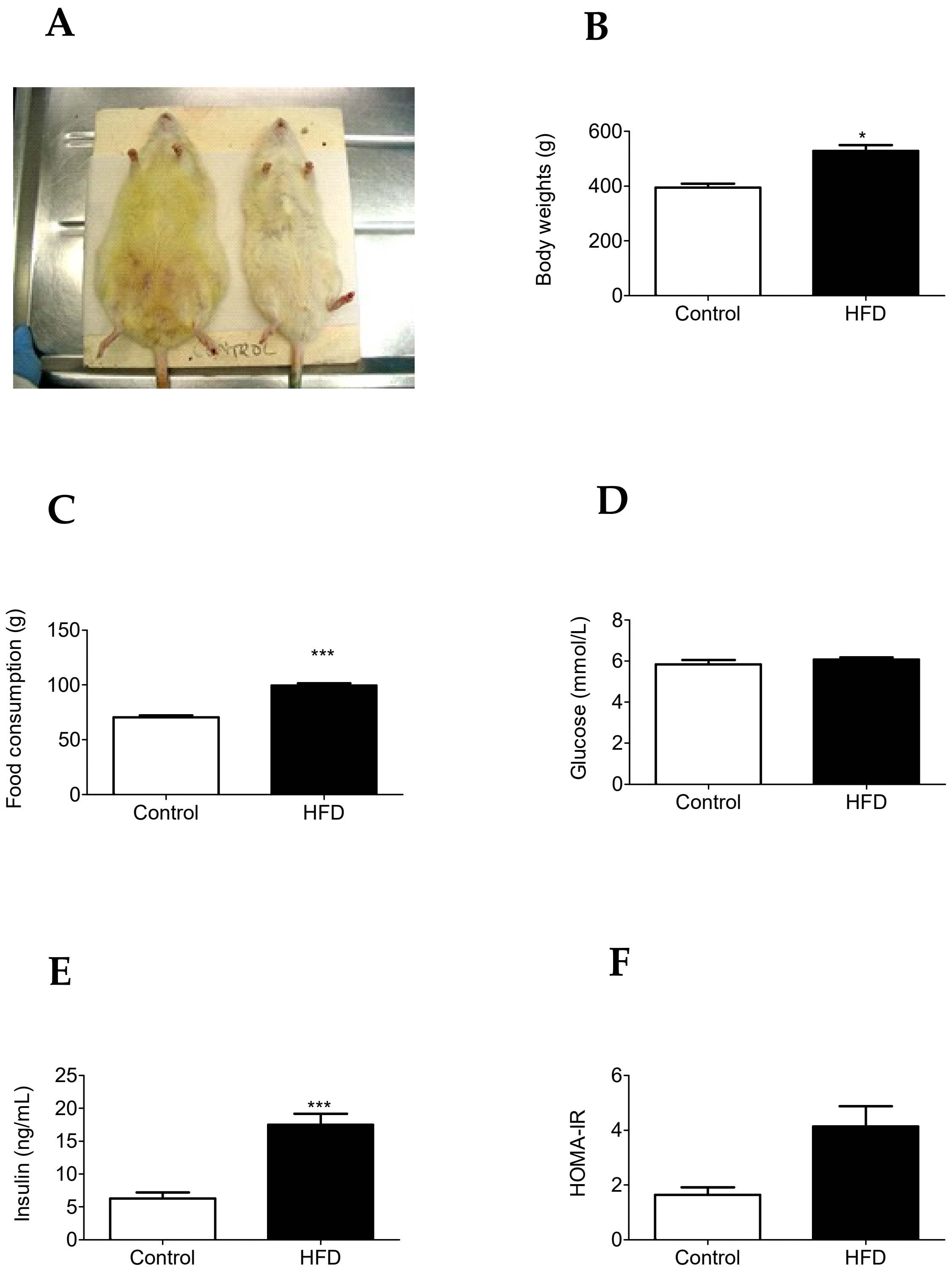

Figure 2S. High fat diet (HFD) induced obese and insulin resistance (OBIR) in male Wistar rats. Graphs shows body weight (A-B), food intake (C), glucose levels (D), insulin (E) and homeostatic model assessment-insulin resistance (HOMA-IR) (F). Wistar rats were treated with or without high fat diet for 12 weeks. Metabolic parameters were measured after 12 weeks. Results are presented as mean ± SEM of Wistar rats (n = 10). * $p < 0.05$; *** $p < 0.001$ versus normal control.

Treatment of OBIR rat model

Since the OBIR model was partially established, in the current study we used only OBIR rats' vs OBIR rats treated with different concentrations of GRE.

Food and water intake in OBIR rats

Twenty-four hour water and food intake were determined, in metabolic cages, prior to commencement of treatment with Rooibos extract as well as 12 weeks after treatment with GRE. Oral glucose tolerance test (OGTT) was performed after 12 weeks of treatment with GRE. After 16 h of fast, rats were gavaged with GRE treatments 1 h (t = -60 min) before administration of a glucose bolus (t = 0 min) at 2 g/kg glucose (50% Dextrose-Fresenius 50%). Plasma glucose concentrations were determined at -60 and at 0, 15, 30, 60, 120 and 240 min, respectively, relative to the glucose bolus (t = 0)

Results

Food consumption

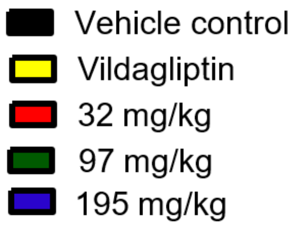

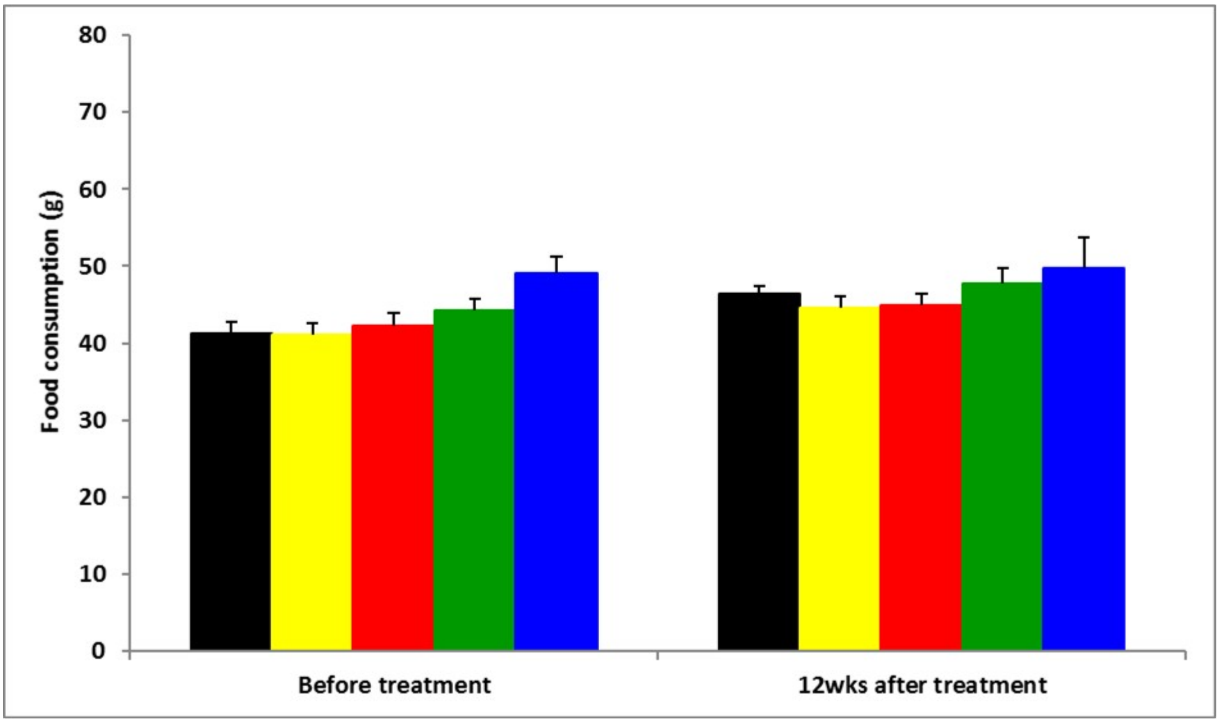

Figure 3S. Obese insulin resistant (OBIR) Wistar rats treated with an aspalathin-enriched green rooibos extract (GRE). Graphs depict food intake before and after 12-week treatment period. The effect of different doses of GRE (32, 97 and 195 mg/kg BW) were assessed for their effect on food intake. No significant results were observed within groups before and after treatment. Results are presented as mean ± SEM of OBIR rats (n = 12).

Water consumption

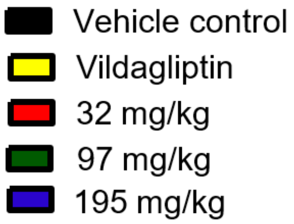

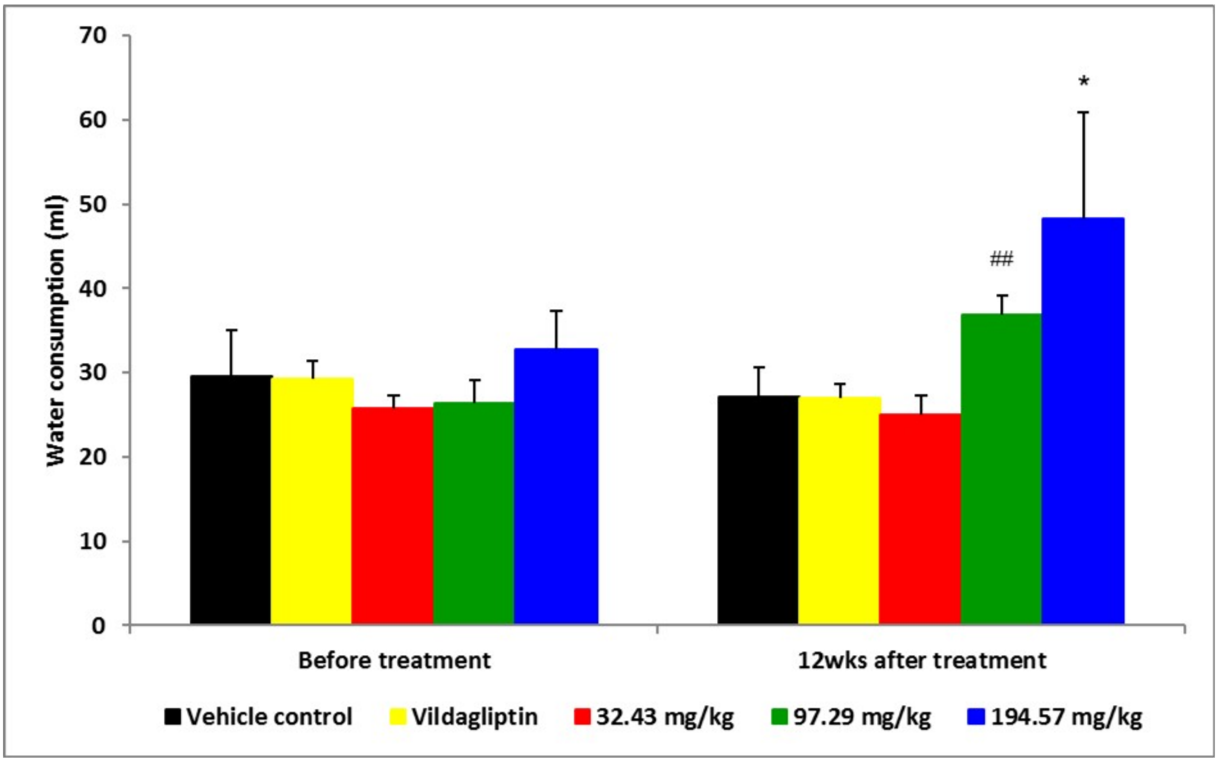

Figure 4S. Obese insulin resistant (OBIR) Wistar rats treated with an aspalathin-enriched green rooibos extract (GRE). Graphs depict water intake before and after 12-week treatment period. The effect of different doses of GRE (32, 97 and 195 mg/kg BW) were assessed for their effect on water intake. The highest dose (194.57 mg/kg/day) significantly increased water intake compared to the baseline controls. Treatment group with 97.29 mg/kg/day displayed significantly increased water intake compared to its baseline values. Results are presented as mean ± SEM of OBIR rats (n = 12). * $p \leq 0.05$ versus respective baseline values; ## $p < 0.01$ versus its baseline values at 12 weeks after treatment. Baseline indicates measurements taken before treatment.

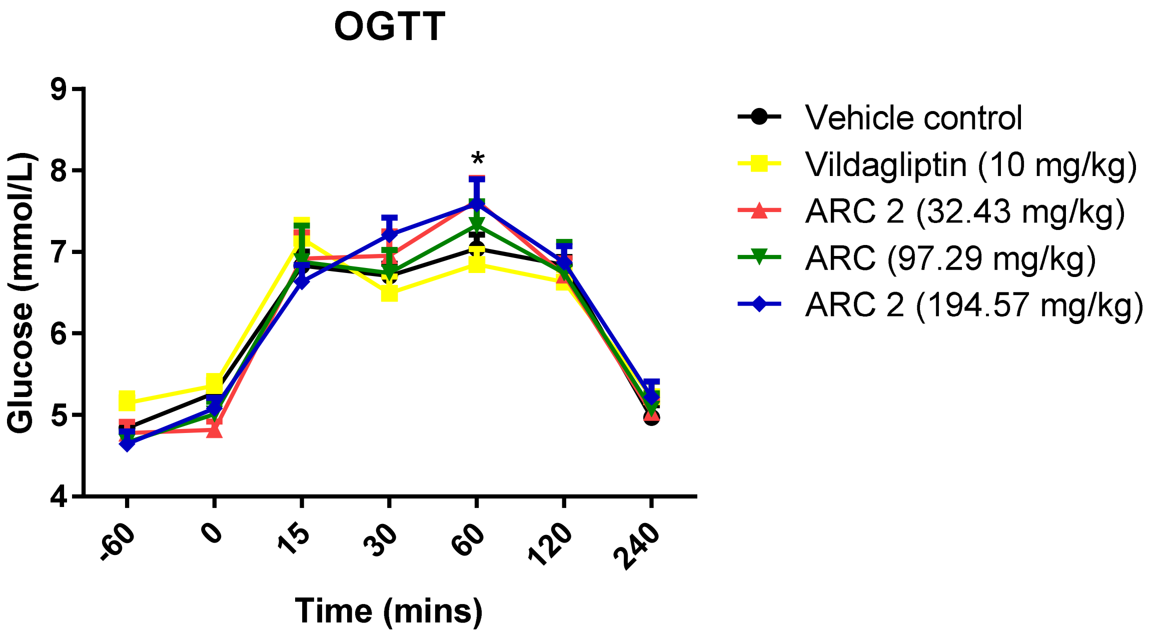

Figure 5S. Obese insulin resistant (OBIR) Wistar rats treated with an aspalathin-enriched green rooibos extract (GRE). Graphs shows oral glucose tolerance test (OGTT) after 12 weeks of treatment with GRE and after 16 h of fast. The effect of different doses of GRE (32, 97 to 195 mg/kg BW) were assessed for their effect on OGTT after 16 hours of fast) * $p \le 0.05$ significance between Vildagliptin and GRE (194.57).

© 2018 by the authors. Submitted for possible open access publication under the terms and conditions of the Creative Commons Attribution (CC BY) license (http://creativecommons.org/licenses/by/4.0/).
